# Screening for trauma‐related symptoms via a smartphone app: The validity of Smart Assessment on your Mobile in referred police officers

**DOI:** 10.1002/mpr.1579

**Published:** 2017-09-25

**Authors:** Christianne A.I. van der Meer, Anne Bakker, Bart A.L. Schrieken, Marthe C. Hoofwijk, Miranda Olff

**Affiliations:** ^1^ Academic Medical Centre, Department of Psychiatry University of Amsterdam Amsterdam The Netherlands; ^2^ Interapy Amsterdam The Netherlands; ^3^ Centre for Psychological Trauma Diemen The Netherlands; ^4^ Arq Psychotrauma Expert Group Diemen The Netherlands

**Keywords:** mHealth, posttraumatic stress disorder, screening, smartphone applications, trauma‐related symptoms

## Abstract

To facilitate easily accessible screening for trauma‐related symptoms, a web‐based application called Smart Assessment on your Mobile (SAM) was developed. In this study, we examined whether SAM was able to accurately identify posttraumatic stress disorder (PTSD) and depression in adults. Eighty‐nine referred police officers completed SAM, containing the PTSD Checklist for *Diagnostic and Statistical Manual of Mental Disorders* (DSM)‐5 (PCL‐5) and the Depression Anxiety and Stress Scale (DASS‐21), on their own device prior to a diagnostic interview where the Clinician‐Administered PTSD Scale for DSM‐5 (CAPS‐5) and Structured Clinical Interview for DSM‐IV (SCID‐I/P) were administered. Results showed a substantial agreement between SAM and the diagnostic interview in the assessment of PTSD and depression. An optimal trade‐off between sensitivity (89%) and specificity (68%) levels was found at a cut‐off score of 31 on the PTSD Checklist for DSM‐5 (area under the curve = 0.845, 95% CI [0.765, 0.925], diagnostic odds ratio = 15.97). This is one of the first studies to support the validity and reliability of a mobile screener following trauma. SAM may facilitate screening for trauma‐related symptoms on a large scale and could be a first step in a stepped‐care model for trauma survivors to help identify individuals who need further diagnostics and care.

## 1 INTRODUCTION

It is widely acknowledged that most of us will be confronted with a potential traumatic event (PTE) during our life, such as loss, serious injuries, or (sexual) violence (Benjet et al., 2016; Breslau, 2009; de Vries & Olff, 2009; Kilpatrick et al., 2013). After experiencing a PTE, individuals are at increased risk to develop profound psychological problems, such as posttraumatic stress disorder (PTSD) and depression (Benjet et al., 2016; Breslau, 2009; de Vries & Olff, 2009). PTSD and depression are both listed at the top of the most common psychiatric disorders (Kessler, Petukhova, Sampson, Zaslavsky, & Wittchen, 2012; O'Donnell, Creamer, & Pattison, 2004; Shalev et al., 1998). Despite the fact that a considerable proportion of people suffer from these psychological problems after trauma, the majority of people do not seek professional help, which can lead to deterioration and undertreatment of these symptoms (Bijl & Ravelli, 2000; Brackbill, Stellman, Perlman, Walker, & Farfel, 2013; Brewin et al., 2010; Graves et al., 2011; Grubaugh et al., 2005; Shalev, Ankri, Peleg, Israeli‐Shalev, & Freedman, 2011).

This treatment gap is unfortunate because effective treatments for trauma‐related symptoms exist (Bisson & Andrew, 2007; Cusack et al., 2016). Moreover, it has been proven that the sooner individuals receive treatment after trauma, the fewer symptoms will arise (Roberts, Kitchiner, Kenardy, & Bisson, 2009). Therefore, it is of crucial importance to (timely) detect trauma‐related symptoms, such as PTSD and depression, and refer individuals who need professional care as soon as possible. This is however a great challenge. Besides personal factors such as embarrassment, avoidant behaviour, and the fear of stigma (Corrigan, 2004), regular health care institutions frequently do not have the capacity and resources to reach all trauma survivors and identify the ones who need further diagnostics or care (Brewin et al., 2010; Shalev et al., 2011). Easily accessible and low‐cost screening tools could make an important contribution to the (early) detection and appropriate referral of individuals with mental health needs in the aftermath of trauma (Grubaugh et al., 2005; Olff, 2015; Price, Kuhn, Hoffman, Ruzek, & Acierno, 2015; Price, Sawyer, Harris, & Skalka, 2016; Price, Yuen, et al., 2014).

Currently, there is an explosive worldwide growth in smartphone and applications (apps) usage (Donker et al., 2013; Olff, 2015), which offers new possibilities in reaching and delivering care to a wide range of people. Mobile health (mHealth) is a specific, upcoming field that focuses on using apps to improve medical or mental health care (Istepanaian & Zhang, 2012; Olff, 2015; Price, Yuen, et al., 2014). Mobile health provides a great opportunity to enhance the process of (timely) detection of trauma‐related symptoms, both after personal, small‐scale traumatic events and after major disasters (Donker et al., 2013; Olff, 2015; Price, Yuen, et al., 2014). Via apps, easily accessible and low‐cost trauma‐related screening instruments can be delivered, which may help individuals recognize symptoms, stimulate help‐seeking behaviour, and contribute to appropriate referral (Brewin et al., 2010; Bush, Skopp, Smolenski, Crumpton, & Fairall, 2013; Olff, 2015; Price, Ruggiero, et al., 2014; Price, Yuen, et al., 2014; Price et al., 2016).

Although the great potential of mHealth tools in improving (mental) health care is widely recognized, research on the validity, reliability, and effectiveness of these tools is rarely conducted and described (Donker et al., 2013; Olff, 2015). In comparison to the increased knowledge on electronic health (eHealth), scientific support for mHealth tools is considerably lacking behind (Donker et al., 2013). Donker et al. (2013) reviewed the empirical literature on mobile apps in general mental health care and identified nearly 5,000 studies mainly describing the development and content of the apps. Strikingly, the majority of these studies did not address the validity, reliability, or efficacy of the apps. Finally, only eight studies describing five different apps targeting depression, stress, and substance use were included in the review as they were the only studies that examined the efficacy of the tools, included a preassessment and postassessment, and described psychological outcome measures. Of these five apps, four were efficacious in reducing psychological problems (Donker et al., 2013). In addition, some studies have been conducted on mobile apps that screen for the general mental health (BinDhim et al., 2015; Bush et al., 2013), but none of these were specifically aimed at trauma survivors. Regarding eHealth interventions in general and in the psychotrauma field, scientific support is growing and results are promising. For instance, a meta‐analysis on the scientific evidence for eHealth targeting PTSD symptoms showed that Internet‐based therapies (i.e., cognitive–behavioural therapy and expressive writing) were more efficacious in reducing PTSD symptoms than control conditions (i.e., wait list, psychoeducation, and control writing task) (Donker et al., 2013; Kuester, Niemeyer, & Knaevelsrud, 2016).

Up to now, only a few studies have been performed on the feasibility and usability of mobile devices for assessing mental health after trauma (Donker et al., 2013; Olff, 2015; Price, Ruggiero, et al., 2014; Price et al., 2015, 2016). Notably, the previous studies mainly focused on the tools' feasibility and usability. Regarding monitoring, Price, Yuen, et al. (2014) examined the feasibility of monitoring mental health via daily text messages in patients who recently experienced a traumatic injury. Results showed that the satisfaction with and response rate to this mobile monitoring system were high and that text messaging could be a useful and efficient method to communicate with traumatic injury patients, and to monitor their mental health (Price, Ruggiero, et al., 2014). In another study, the usability of a mobile app to monitor posttrauma symptoms in adults with a trauma history was investigated (Price et al., 2016). The participants indicated that usage of the app could improve their recovery from the traumatic event and they preferred an app that would provide immediate feedback on their mental health status, was easy to use, and was customizable (Price et al., 2016). In addition, Price et al. (2015) investigated whether responses to a self‐report measure on PTSD administered via a mobile device differed from paper administration in a sample of trauma‐exposed adults. No differences were found in total and item scores on the PTSD self‐report measure between the two administration methods (Price et al., 2015). Importantly, usability and feasibility results from the available studies are very promising, but so far, there is no evidence for the validity and reliability of mHealth screening tools following trauma.

In order to address the abovementioned concerns and to facilitate screening for trauma‐related symptoms, we designed a web‐based app called Smart Assessment on your Mobile (SAM). SAM comprises modules to assess several relevant mental health domains that may be affected after trauma, as well as well‐known risk and protective factors that influence mental health. As a first step to examine the validity of SAM, we investigated whether SAM was able to correctly identify PTSD and depression in a sample of referred police officers. We hypothesized that SAM would be a valid (diagnostic) screener and therefore would be able to accurately assess PTSD and depression.

## 2 METHODS

### 2.1 Participants

The sample consisted of trauma‐exposed police officers who were referred to the police outpatient clinic for a diagnostic interview. The period between the experienced traumatic event and the referral (and therefore the diagnostic interview) was at least one month. Between November 2014 and July 2015, 113 individuals were referred to the outpatient clinic, of whom 94 (83.2%) consented to participate. Five participants (5.3%) did not complete the PTSD Checklist for *Diagnostic and Statistical Manual of Mental Disorders* (DSM)‐5 (PCL‐5) within SAM and were omitted from the analyses, leaving the data of 89 participants (88 police officers and one ambulance worker) for statistical analyses.

The 89 participants, of whom 67 were men (75.3%), had a mean age of 44.8 years (*SD* = 12.25, range 21–67). Most of the participants (87.6%) had a partner, and 80.9% reported completing a medium educational level (comparable to a college degree). Thirty‐nine participants (43.8%) reported that they were currently being treated for psychological problems, of whom 29 (74.4%) indicated that this concerned treatment for trauma‐related issues (as determined within SAM). During the diagnostic interview, participants could indicate multiple experienced traumatic events. The most commonly experienced types of traumatic events were all work related: witnessing a deceased person (44.6%), witnessing a severe accident (37.3%), and being confronted with violence (28.9%).

### 2.2 Procedure

All participants were scheduled for a diagnostic interview at the police outpatient clinic for the assessment of trauma‐related symptoms. The police outpatient clinic is an independent national centre in the Netherlands where clinical diagnosticians, psychologist, and psychiatrists are specialized in investigating trauma‐related symptoms in police officers (van der Meer et al., 2016). Researchers contacted the participants by telephone prior to their diagnostic interview to provide information about the study aim and procedures. The researchers explained by phone that the aim of the study was to investigate if a mobile application was able to correctly assess mental health and identify trauma‐related symptoms after stressful events. It was explained that the mobile application contained questions about mental health, particularly following trauma, and that the app did not provide feedback on mental health status. Participants were asked to complete SAM once, prior to their scheduled diagnostic interview, on their own personal device. Participants could decide when and where they wanted to fill in SAM.

Participants were informed that all obtained data would be treated as strictly confidential and that only the researchers would have access to the data. The clinical diagnosticians at the police outpatient clinic were blind to the participants' results in SAM. If a person orally consented to participate in the study, the researchers e‐mailed a digital study information brochure and a web link to open SAM. Participants were asked to complete all the questions within SAM in one session. The mean time between completing SAM and the diagnostic interview was 5.6 days (*SD* = 4.6). Of the 89 participants, 76.4% completed SAM within a week prior to the diagnostic interview. This study was conducted in compliance with ethical principles. The Medical Ethical Committee of the Academic Medical Centre exempted this study from formal review.

### 2.3 Measures

#### 2.3.1 Smart Assessment on Your Mobile

SAM is a web‐based app that can be used on a smartphone, PC, or tablet. SAM assesses several mental health domains that might be affected after PTEs, including PTSD symptoms, general functioning, depression, anxiety, stress, work engagement, substance use, and physical functioning. In addition, SAM evaluates well‐known risk factors (trauma exposure and peritraumatic reactions) as well as protective factors (social support and psychological resilience) that may influence these mental health outcomes. To ensure confidentiality, the obtained data within SAM were encrypted before they were sent from the participant's device to a secured database. SAM did not provide feedback to the participants about their mental health status or symptom severity. The domains within SAM are measured with freely available questionnaires. Each questionnaire was presented on a separate screen and was introduced by using the official introduction text of the original scale. Participants were provided with the contact details of the researchers in case of any (technical) problems.

As a first step in validating SAM, this study addresses the accuracy of SAM in assessing PTSD and depressive symptoms after trauma exposure. For the assessment of PTSD symptoms, SAM contains the Dutch version of the PCL‐5 without the criterion A component (Boeschoten, Bakker, Jongedijk, & Olff, 2014; Weathers et al., 2013). In this version, criterion A is not specifically assessed and the PCL‐5 scores are not related to a specific event. The PCL‐5 is a widely used self‐report questionnaire assessing the 20 DSM‐5 PTSD symptoms in the past month. The four measured PTSD symptom clusters are cluster B *reexperiencing* (five items), cluster C *avoidance* (two items), cluster D *negative alterations in cognitions and mood* (seven items), and cluster E *hyperarousal* (six items). Each item is rated on a 5‐point Likert scale, ranging from 0 (*not at all*) to 4 (*extremely*), resulting in a total score ranging from 0 to 80. A certain symptom is present when the PCL‐5 score is 2 (*moderately*) or higher. An indication for possible PTSD based on PCL‐5 scores may be derived in two ways: (a) by applying the DSM‐5 diagnostic rule, which requires the presence of ≥ 1 symptom in cluster B and C and ≥ 2 symptoms in cluster D and E, or 2) by applying the currently suggested cut‐off point of ≥ 33 (http://www.ptsd.va.gov/professional/assessment/adult-sr/ptsd-checklist.asp). Several studies have found good psychometric properties for the PCL‐5, with good internal consistency (ranging from 0.56 to 0.96 for the total scale), test–retest reliability, convergent validity, discriminant validity, and diagnostic utility (Armour, Contractor, Shea, Elhai, & Pietrzak, 2016; Ashbaugh, Houle‐Johnson, Herbert, El‐Hage, & Brunet, 2016; Blevins, Weathers, Davis, Witte, & Domino, 2015; Bovin et al., 2016; Wortmann et al., 2016). The internal consistency in the current study was excellent for the PCL‐5 total severity score (α = .93) and good for all subscales: reexperiencing (α = .87), avoidance (α = .84), negative alterations in cognitions and mood (α = .83), and hyperarousal (α = .76).

To evaluate depressive symptoms, SAM contains the short Dutch version of the well‐established Depression Anxiety and Stress Scale (DASS‐21; de Beurs, Van Dyck, Marquenie, Lange, & Blonk, 2001). Participants indicated to what extent the statements applied to them on a 4‐point Likert scale, ranging from 0 (*not at all*) to 3 (*very often*). The depression subscale consists of seven items, and each score has to be multiplied by 2 to calculate the final score. A score between 21 and 27 on the depression subscale indicates severe depressive symptoms. The instrument has good reliability and good test–retest reliability (de Beurs et al., 2001). Internal consistency for the depression subscale in the current sample was excellent (α = .91).

Concerning demographics and psychiatric history, SAM asks for gender, age, education, and marital status. Furthermore, participants indicated if they were treated (currently) for psychological problems and, if so, for what kind of psychological problems.

#### 2.3.2 Diagnostic interview

The clinical diagnostician of the police outpatient clinic conducted an unstructured interview with the participant about the current psychological symptoms and the potential causes and event(s) that led to the development of these symptoms. Thereafter, the clinical diagnostician determined the presence of a traumatic event according to criterion A. Criterion A requires an exposure to actual or threatened death, serious injury, or sexual violence by experiencing it, personally witnessing it, learning that the event(s) occurred to a close family member or close friend, and/or experiencing repeated or extreme exposure to aversive details of the event(s) (American Psychiatric Association, 2013). If more than one traumatic event was experienced, the clinical diagnostician asked which of the experienced events currently had the most negative impact on the participant's life (index trauma). Each index trauma was categorized by the researchers into work‐related versus private events.

During the diagnostic interview, the golden standard Clinician‐Administered PTSD Scale for DSM‐5 (CAPS‐5) was used to measure PTSD symptoms in the past month and to determine DSM‐5 diagnosis for PTSD (Boeschoten et al., 2014; Weathers et al., 2013). The clinical diagnostician only administered the CAPS‐5 when Criterion A was met. The CAPS‐5 is a 30‐item structured clinical interview. Items are rated with a single severity score, ranging from 0 (*absent*) to 4 (*extreme or incapacitating*). A symptom is considered endorsed if the severity score is 2 (*moderate or threshold*) or higher. The total symptom severity score (range 0–80) is determined by summing up the severity scores of all 20 DSM‐5 symptoms. A PTSD diagnosis is established by following the diagnostic rule for DSM‐5 symptom criteria. Furthermore, the disturbances have to last at least one month (criterion F) and should cause either clinically significant distress or functional impairment (criterion G). In this study, the internal consistency was good for the CAPS‐5 total severity score (α = .90) and all subscales: re‐experiencing (α = .82), avoidance (α = .66), negative alterations in cognitions and mood (α = .76), and hyperarousal (α = .76).

To determine Axis I diagnoses other than PTSD, the Dutch version (Van Groenestijn, Akkerhuis, Kupka, Schneider, & Nolen, 1999) of the Structured Clinical Interview for DSM‐IV (SCID‐I/P; Spitzer, Gibbon, Janet, & Janet, 1996) was administered. The SCID‐I/P measures several Axis I disorders, including mood disorders.

### 2.4 Statistical analyses

The internal consistency was determined by calculating Cronbach's alpha for PCL‐5 and CAPS‐5 total and subscale scores and the DASS‐21 depression subscale. Further, we calculated descriptive statistics for age, gender, educational level, current psychological treatment, traumatic events, PTSD symptoms (PCL‐5 and CAPS‐5), and PTSD diagnosis (CAPS‐5).

To examine the agreement between the indication for possible PTSD in SAM (PCL‐5) and the clinician‐derived PTSD diagnosis (CAPS‐5), the following statistics were calculated between the two measurements: the number of true positives (individuals with PTSD as assessed with the CAPS‐5 correctly identified by the PCL‐5 in SAM), true negatives (individuals without PTSD correctly identified as non‐PTSD cases in SAM), false positives (individuals without PTSD incorrectly identified as PTSD cases in SAM), and false negatives (individuals with PTSD incorrectly identified as non‐PTSD cases in SAM); the observed agreement (*p*_o_; percentage of agreement between the PCL‐5 and CAPS‐5); and kappa (κ; measure of the magnitude of the observed agreement). In addition, convergent validity was investigated by calculating Spearman's rho correlations (non‐normally distributed variables) between the PCL‐5 and CAPS‐5 total and subscale scores.

To investigate the diagnostic accuracy for PTSD symptoms of the PCL‐5 in SAM, a receiver operating characteristic curve was calculated and the sensitivity (the proportion of people with PTSD according to the CAPS‐5 correctly identified in SAM), specificity (the proportion of people without PTSD correctly identified in SAM), optimal cut‐off point, and the area under the curve (AUC) were determined. Also, the diagnostic odds ratio (DOR; ratio between true positives and true negatives) for the optimal cut‐off point on the PCL‐5 was calculated.

Finally, to examine the comparability between depressive symptoms in SAM (DASS‐21 depression subscale) and the clinician‐derived depression diagnosis (SCID‐I/P), the observed agreement, kappa, and DOR values were calculated. All analyses were performed in IBM SPSS Statistics Version 23. A *p* value of <.05 was considered significant.

## 3 RESULTS

### 3.1 Posttraumatic stress disorder symptoms

Fifty‐two (58.4%) participants were diagnosed with PTSD according to the CAPS‐5 and had an average CAPS‐5 total severity score of 34.88 (*SD* = 9.36, range 18–64). Within SAM, in 56 (62.9%) and 54 (60.7%) participants, the PCL‐5 scores indicated possible PTSD, according to the diagnostic rule and the suggested cut‐off of 33, respectively. The observed agreement between the CAPS‐5 and the PCL‐5 in SAM was 77.5% (κ = .530, diagnostic rule) and 75.3% (κ = .487, suggested cut‐off of 33), indicating a moderate interrater agreement. The DOR of the PCL‐5 in SAM was 11.46 (diagnostic rule) and 8.750 (cut‐off of 33). Participants with a clinician‐rated PTSD diagnosis had significantly higher scores on the PCL‐5 (*M* = 43.10, *SD* = 13.55) than participants without a PTSD diagnosis (*M* = 25.73, *SD* = 11.01), *t*(87) = −6.43, *p* < .001.

### 3.2 Convergent validity for posttraumatic stress disorder symptoms

The PCL‐5 total score showed a significant positive correlation with the total CAPS‐5 score (*r*_s_ = .768, *p* < .001). This applied as well to the separate subscales: reexperiencing (*r*_s_ = .718, *p* < .001), avoidance (*r*_s_ = .537, *p* < .001), negative alterations in cognitions and mood (*r*_s_ = .683, *p* < .001), and hyperarousal (*r*_s_ = .620, *p* < .001).

### 3.3 Diagnostic accuracy for posttraumatic stress disorder symptoms

**Figure 1:**
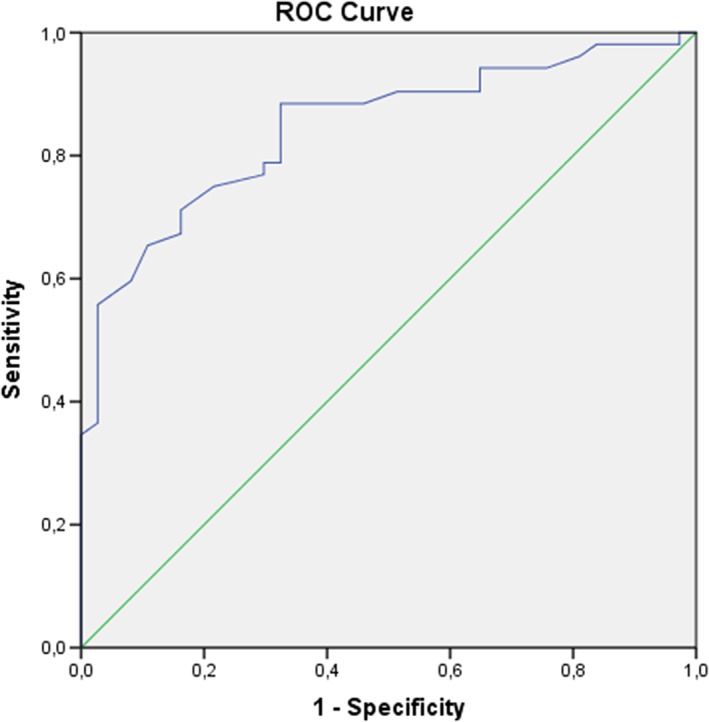
Receiver operating characteristic (ROC) curve for the PCL‐5

The receiver operating characteristic curve for the PCL‐5 is shown in Figure 1. The AUC of the PCL‐5 was 0.845 (95% CI [0.765, 0.925]). The sensitivity of the PCL‐5 was 81%, and the specificity was 68% at the cut‐off of 33. The optimal balance between sensitivity and specificity for this study population was found at a cut‐off of 31, with a sensitivity of 89%, a specificity of 68%, and an observed agreement of 79.8% (κ = .574). The DOR of the PCL‐5 at the cut‐off of 31 was 15.97, indicating that participants who score ≥ 31 have a 15.97 higher chance to have PTSD than participants who score < 31 on the PCL‐5. Table 1 shows an overview of the true‐positive, true‐negative, false‐positive, and false‐negative values for the PCL‐5.

**Table 1:** TP, TN, FP, FN, and total correctly identified cases for the PCL‐5 within SAM (*n* = 89)

	TP	TN	FP	FN	Total correctly identified cases, *n* (%)
PCL‐5 diagnostic criteria	44	25	12^a^	8	69 (77.5)
PCL‐5 ≥ 33	42	25	12^a^	10	67 (75.3)
PCL‐5 ≥ 31	46	25	12^a^	6	71 (79.8)

*Note*. FN = false negatives; FP = false positives; PCL‐5 = Posttraumatic Stress Disorder Checklist for *Diagnostic and Statistical Manual of Mental Disorders* 5; SAM = Smart Assessment on your Mobile; TN = true negatives; TP = true positives. Total correctly identified cases = TP + TN.

a In two of these 12 participants, the experienced event did not meet criterion A (as determined during the diagnostic interview); therefore, the Clinician‐Administered Posttraumatic Stress Disorder Scale for *Diagnostic and Statistical Manual of Mental Disorders* 5 (CAPS‐5) was not administered beyond criterion A and posttraumatic stress disorder was not diagnosed.

### 3.4 Depressive symptoms

According to the SCID at intake, 19 (21.3%) participants were diagnosed with depression. Regarding the DASS‐21 depression subscale within SAM, in 20 (22.5%) participants, the scores indicated severe depressive symptoms. The agreement between the SCID and the DASS‐21 depression subscale in SAM was 76.4% (κ = .328), indicating fair interrater agreement. The DOR of the DASS‐21 depression subscale was 5.31. Participants with a clinician‐rated depressive disorder had significantly higher scores on the DASS‐21 depression subscale (*M* = 10.84, *SD* = 4.02) than participants without a depressive disorder (*M* = 6.14, *SD* = 4.46), *t*(86) = −4.15, *p* < .001.

## 4 DISCUSSION

The current study is one of the first studies to report on the validity of a mobile screener in the aftermath of trauma. The results show that SAM was able to accurately screen for PTSD and depressive symptoms and therefore can be considered a valid and reliable (diagnostic) screening tool in the investigated population.

Overall, our results revealed a substantial degree of agreement between SAM and the diagnostic interview in the assessment of PTSD symptoms and depressive symptoms. There was a strong, positive association between the PCL‐5 in SAM and CAPS‐5 interview total scores. Similarly, the separate symptom clusters reexperiencing, avoidance, negative alterations in cognitions and mood, and hyperarousal (as measured by both instruments) showed significant positive correlations, but the association between the PCL‐5 and CAPS‐5 on the cluster avoidance was relatively low. This could be explained by the fact that the subscale only consists of two items, whereas the other subscales include more items.

An optimal cut‐off score of 31 (with a sensitivity of 89% and a specificity of 68%) was found for the PCL‐5 in SAM for possible PTSD in our sample of referred police officers. This cut‐off is slightly lower than the currently suggested cut‐off of 33 (http://www.ptsd.va.gov/professional/assessment/adult-sr/ptsd-checklist.asp), but in line with the results of a recent study that validated the PCL‐5 against the CAPS‐5 in veterans from a large Veterans Affairs Health Care database (Bovin et al., 2016). Also, a study in undergraduate students showed an optimal cut‐off point of 31 and 32 for the French and English versions of the PCL‐5, respectively (however, this study did not validate the PCL‐5 against a DSM‐5 golden standard such as the CAPS‐5; Ashbaugh et al., 2016). In addition, the sensitivity level of 89% in our current study clearly exceeds the recommended minimal sensitivity level of 80% for PTSD screening instruments (Mouthaan, Sijbrandij, Reitsma, Gersons, & Olff, 2014; O'Donnell, Bryant, Creamer, & Carty, 2008). Moreover, our AUC value of 0.845 confirmed that the PCL‐5 in SAM adequately differentiates between individuals with and without PTSD.

Besides PTSD symptoms, SAM was also able to correctly identify depressive symptoms. Results showed a satisfactory agreement between the self‐reported DASS‐21 depression subscale within SAM and the SCID during the diagnostic interview. The overall agreement, interrater agreement, and DOR values were slightly lower for depressive symptoms than for PTSD symptoms.

Interestingly, a remarkably high response rate (83.2%) and even higher completion rate (94.7%) were found in our study. This may imply that the willingness to use SAM was high and the app was easy to use. Regarding the willingness to use mobile screeners for mental health, one of the few studies on a mobile screener for mental health found that active‐duty soldiers highly preferred their smartphone over computer and paper methods to complete screening measures (Bush et al., 2013). Also, a study on a screening app for depression showed that a large number of people from different countries downloaded the app, and the majority (73.9%) of these individuals completed the depression questionnaire within the app (BinDhim et al., 2015). Although speculative, these findings suggest that the potential uptake and usage of mobile screeners for mental health could be high.

Mobile screeners like SAM may reach a diverse range of trauma‐exposed individuals, both in the general population and in high‐risk professions where employees are continuously exposed to PTEs (van der Meer et al., 2016). These tools may be incorporated as a first step in a (cost‐)effective stepped‐care model to help identify individuals who need further diagnostic examination and care (Price et al., 2016). Further developments may enhance the added value and uptake of mobile screeners in the field of trauma. Incorporating personalized feedback to the end‐user is recommended to increase the usability and sustained use of posttrauma mobile screeners (Price et al., 2016). In addition, these tools would be even more valuable if they provide contact details of professional health care institutions (Olff, 2015; Price et al., 2016) and are linked to other evidence‐based mobile apps, such as the PTSD Coach that offers self‐help to individuals with PTSD symptoms (Kuhn et al., 2014; Miner et al., 2016; Owen et al., 2015; Possemato, Kuhn, Johnson, Hoffman, & Brooks, 2016). In this manner, the needs of trauma survivors can be (timely) met and the appropriate posttrauma care can be delivered and received (Olff, 2015; Owen et al., 2015; Price, Ruggiero, et al., 2014; Price, Yuen, et al., 2014; Price et al., 2016).

In a next stage, SAM could provide immediate feedback to the end‐user and may be investigated as a monitoring tool that informs both the end‐user and the clinician about the patient's mental health. SAM is currently implemented at the police outpatient clinic prior to the diagnostic interview to guide clinicians in addressing specific mental health domains that require attention during the diagnostic interview. In the current study, SAM was examined as a diagnostic screener for PTSD; therefore, the period between the experienced traumatic event and usage of SAM was at least one month. Future studies should investigate the validity of SAM in detecting more acute trauma‐related symptoms, such as acute stress disorder (ASD). ASD symptoms are highly similar to PTSD symptoms; however, ASD is only diagnosed within the first month after trauma (American Psychiatric Association, 2013). Prospective studies are needed to examine the use of SAM as a predictive mobile screener in a large sample of individuals who use SAM directly after trauma. Also, further studies should be conducted in a counterbalanced design and study the test–retest reliability of SAM and the sensitivity to clinical change. Lastly, the cost‐effectiveness of SAM and the potential negative consequences of screening need to be addressed in future research endeavours (e.g., the risk of potentially stigmatizing false‐positive cases).

Some limitations of the current study must be considered. Our sample consists of referred (male) police officers, allowing limited generalization to other trauma‐exposed individuals. A proportion of our sample received treatment for trauma‐related issues and was, presumably, familiar with PTSD symptoms. Therefore, these participants were potentially better able to recognize their PTSD symptoms than participants who did not receive treatment for trauma‐related symptoms. This is the first Dutch study to report on PCL‐5 and CAPS‐5 comparison; further studies are necessary to confirm the current findings, also with other (offline) methods, study designs, and other trauma‐exposed populations.

Our study has several important strengths. The response and completion rates in our study were very high, reducing potential biases and the possibility of including a selective subsample. Furthermore, we used well‐established instruments in our study, such as the PCL‐5 and CAPS‐5. Moreover, the diagnostic interview was performed by highly experienced clinical diagnosticians at the police outpatient clinic.

To conclude, this study is a crucial first step in the evaluation of the mobile app SAM and showed that SAM is a valid mobile screener for PTSD and depressive symptoms. Mobile screeners such as SAM can be incorporated as a first step in a stepped‐care model to identify trauma survivors in need of further diagnostics and care. Moreover, SAM may contribute to (timely) referral to appropriate and professional care if needed. It is of utmost importance to conduct high‐quality research on the validity and efficacy of mHealth tools before releasing them into the market (Donker et al., 2013; Olff, 2015). In this manner, we may realize the great opportunities that mHealth offers and provide evidence‐based tools that truly contribute to the improvement of posttrauma care.

## CONFLICT OF INTEREST

The authors have no conflicts of interest to declare.

## References

[mpr1579-bib-0001] American Psychiatric Association (2013). Diagnostic and statistical manual of mental disorders (5th ed.). Washington, DC: American Psychiatric Association.

[mpr1579-bib-0002] Armour, C. , Contractor, A. , Shea, T. , Elhai, J. D. , & Pietrzak, R. H. (2016). Factor structure of the PTSD Checklist for DSM‐5: Relationships among symptom clusters, anger, and impulsivity. The Journal of Nervous and Mental Disease, 204(2), 108–115. 10.1097/NMD.0000000000000430 26669984

[mpr1579-bib-0003] Ashbaugh, A. R. , Houle‐Johnson, S. , Herbert, C. , El‐Hage, W. , & Brunet, A. (2016). Psychometric validation of the English and French versions of the Posttraumatic Stress Disorder Checklist for DSM‐5 (PCL‐5). PloS One, 11(10). e0161645. doi:10.1371/journal.pone.0161645 PMC5056703 27723815

[mpr1579-bib-0004] Benjet, C. , Bromet, E. , Karam, E. G. , Kessler, R. C. , McLaughlin, K. A. , Ruscio, A. M. , … Koenen, K. C. (2016). The epidemiology of traumatic event exposure worldwide: Results from the World Mental Health Survey Consortium. Psychological Medicine, 46(2), 327–343. 10.1017/S0033291715001981 26511595 PMC4869975

[mpr1579-bib-0005] Bijl, R. V. , & Ravelli, A. (2000). Psychiatric morbidity, service use, and need for care in the general population: Results of The Netherlands Mental Health Survey and Incidence Study. American Journal of Public Health, 90(4), 602–607. 10754976 10.2105/ajph.90.4.602 PMC1446190

[mpr1579-bib-0006] BinDhim, N. F. , Shaman, A. M. , Trevena, L. , Basyouni, M. H. , Pont, L. G. , & Alhawassi, T. M. (2015). Depression screening via a smartphone app: Cross‐country user characteristics and feasibility. Journal of the American Medical Informatics Association, 22(1), 29–34. 10.1136/amiajnl-2014-002840 25326599 PMC4433364

[mpr1579-bib-0007] Bisson, J. , & Andrew, M. (2007). Psychological treatment of post‐traumatic stress disorder (PTSD). Cochrane Database of Systematic Reviews, 3. CD003388. doi:10.1002/14651858.CD003388.pub3 17636720

[mpr1579-bib-0008] Blevins, C. A. , Weathers, F. W. , Davis, M. T. , Witte, T. K. , & Domino, J. L. (2015). The Posttraumatic Stress Disorder Checklist for DSM‐5 (PCL‐5): Development and initial psychometric evaluation. Journal of Traumatic Stress, 28(6), 489–498. 10.1002/jts.22059 26606250

[mpr1579-bib-0009] Boeschoten, M. A. , Bakker, A. , Jongedijk, R. A. , & Olff, M. (2014). PTSD checklist for the DSM‐5 (PCL‐5)—Nederlandstalige versie Uitgave. Diemen, The Netherlands: Arq Psychotrauma Expert Groep.

[mpr1579-bib-0010] Boeschoten, M. A. , Bakker, A. , Jongedijk, R. A. , van Minnen, A. , Elzinga, B. M. , Rademaker, A. R. , & Olff, M. (2014). Clinician administered PTSD scale for DSM‐5—Nederlandstalige versie Uitgave. Diemen, The Netherlands: Arq Psychotrauma Expert Groep.

[mpr1579-bib-0011] Bovin, M. J. , Marx, B. P. , Weathers, F. W. , Gallagher, M. W. , Rodriguez, P. , Schnurr, P. P. , & Keane, T. M. (2016). Psychometric properties of the PTSD Checklist for Diagnostic and Statistical Manual of Mental Disorders‐Fifth Edition (PCL‐5) in veterans. Psychological Assessment, 28(11), 1379–1391. 10.1037/pas0000254 26653052

[mpr1579-bib-0012] Brackbill, R. M. , Stellman, S. D. , Perlman, S. E. , Walker, D. J. , & Farfel, M. R. (2013). Mental health of those directly exposed to the World Trade Center disaster: Unmet mental health care need, mental health treatment service use, and quality of life. Social Science & Medicine, 81, 110–114. 10.1016/j.socscimed.2012.12.016 23337833

[mpr1579-bib-0013] Breslau, N. (2009). The epidemiology of trauma, PTSD, and other posttrauma disorders. Trauma Violence Abuse, 10(3), 198–210. 10.1177/1524838009334448 19406860

[mpr1579-bib-0014] Brewin, C. R. , Fuchkan, N. , Huntley, Z. , Robertson, M. , Thompson, M. , Scragg, P. , … Ehlers, A. (2010). Outreach and screening following the 2005 London bombings: Usage and outcomes. Psychological Medicine, 40(12), 2049–2057. 10.1017/s0033291710000206 20178677 PMC2964043

[mpr1579-bib-0015] Bush, N. E. , Skopp, N. , Smolenski, D. , Crumpton, R. , & Fairall, J. (2013). Behavioral screening measures delivered with a smartphone app: Psychometric properties and user preference. The Journal of Nervous and Mental Disease, 201(11), 991–995. 10.1097/NMD.0000000000000039 24177488

[mpr1579-bib-0016] Corrigan, P. (2004). How stigma interferes with mental health care. The American Psychologist, 59(7), 614–625. 10.1037/0003-066x.59.7.614 15491256

[mpr1579-bib-0017] Cusack, K. , Jonas, D. E. , Forneris, C. A. , Wines, C. , Sonis, J. , Middleton, J. C. , … Gaynes, B. N. (2016). Psychological treatments for adults with posttraumatic stress disorder: A systematic review and meta‐analysis. Clinical Psychology Review, 43, 128–141. 10.1016/j.cpr.2015.10.003 26574151

[mpr1579-bib-0018] de Beurs, E. , Van Dyck, R. , Marquenie, L. A. , Lange, A. , & Blonk, R. W. B. (2001). De DASS: Een vragenlijst voor het meten van depressie, angst en stress [the DASS: A questionnaire for depression, anxiety and stress]. Gedragstherapie, 34, 35–53.

[mpr1579-bib-0019] de Vries, G. J. , & Olff, M. (2009). The lifetime prevalence of traumatic events and posttraumatic stress disorder in the Netherlands. Journal of Traumatic Stress, 22(4), 259–267. 10.1002/jts.20429 19645050

[mpr1579-bib-0020] Donker, T. , Petrie, K. , Proudfoot, J. , Clarke, J. , Birch, M. R. , & Christensen, H. (2013). Smartphones for smarter delivery of mental health programs: A systematic review. Journal of Medical Internet Research, 15(11). e247. doi:10.2196/jmir.2791 PMC3841358 24240579

[mpr1579-bib-0021] Graves, R. E. , Freedy, J. R. , Aigbogun, N. U. , Lawson, W. B. , Mellman, T. A. , & Alim, T. N. (2011). PTSD treatment of African American adults in primary care: The gap between current practice and evidence‐based treatment guidelines. Journal of the National Medical Association, 103(7), 585–593. 21999033 10.1016/s0027-9684(15)30384-9

[mpr1579-bib-0022] Grubaugh, A. L. , Magruder, K. M. , Waldrop, A. E. , Elhai, J. D. , Knapp, R. G. , & Frueh, B. C. (2005). Subthreshold PTSD in primary care: Prevalence, psychiatric disorders, healthcare use, and functional status. The Journal of Nervous and Mental Disease, 193(10), 658–664. 16208161 10.1097/01.nmd.0000180740.02644.ab

[mpr1579-bib-0023] Istepanaian, R. S. , & Zhang, Y. T. (2012). Guest editorial. Introduction to the special section: 4G health—The long‐term evolution of m‐Health. IEEE Transactions on Information Technology in Biomedicine, 16(1), 1–5. 10.1109/TITB.2012.2183269 22271836

[mpr1579-bib-0024] Kessler, R. C. , Petukhova, M. , Sampson, N. A. , Zaslavsky, A. M. , & Wittchen, H. U. (2012). Twelve‐month and lifetime prevalence and lifetime morbid risk of anxiety and mood disorders in the United States. International Journal of Methods in Psychiatric Research, 21(3), 169–184. 10.1002/mpr.1359 22865617 PMC4005415

[mpr1579-bib-0025] Kilpatrick, D. G. , Resnick, H. S. , Milanak, M. E. , Miller, M. W. , Keyes, K. M. , & Friedman, M. J. (2013). National estimates of exposure to traumatic events and PTSD prevalence using DSM‐IV and DSM‐5 criteria. Journal of Traumatic Stress, 26(5), 537–547. 10.1002/jts.21848 24151000 PMC4096796

[mpr1579-bib-0026] Kuester, A. , Niemeyer, H. , & Knaevelsrud, C. (2016). Internet‐based interventions for posttraumatic stress: A meta‐analysis of randomized controlled trials. Clinical Psychology Review, 43, 1–16. 26655959 10.1016/j.cpr.2015.11.004

[mpr1579-bib-0027] Kuhn, E. , Greene, C. , Hoffman, J. , Nguyen, T. , Wald, L. , Schmidt, J. , … Ruzek, J. (2014). Preliminary evaluation of PTSD Coach, a smartphone app for post‐traumatic stress symptoms. Military Medicine, 179(1), 12–18. 10.7205/MILMED-D-13-00271 24402979

[mpr1579-bib-0028] Miner, A. , Kuhn, E. , Hoffman, J. E. , Owen, J. E. , Ruzek, J. I. , & Taylor, C. B. (2016). Feasibility, acceptability, and potential efficacy of the PTSD Coach app: A pilot randomized controlled trial with community trauma survivors. Psychol Trauma, 8(3), 384–392. 10.1037/tra0000092 27046668

[mpr1579-bib-0029] Mouthaan, J. , Sijbrandij, M. , Reitsma, J. B. , Gersons, B. P. , & Olff, M. (2014). Comparing screening instruments to predict posttraumatic stress disorder. PloS One, 9(5). e97183. doi:10.1371/journal.pone.0097183 PMC4016271 24816642

[mpr1579-bib-0030] O'Donnell, M. L. , Bryant, R. A. , Creamer, M. , & Carty, J. (2008). Mental health following traumatic injury: Toward a health system model of early psychological intervention. Clinical Psychology Review, 28(3), 387–406. 10.1016/j.cpr.2007.07.008 17707563

[mpr1579-bib-0031] O'Donnell, M. L. , Creamer, M. , & Pattison, P. (2004). Posttraumatic stress disorder and depression following trauma: Understanding comorbidity. The American Journal of Psychiatry, 161(8), 1390–1396. 10.1176/appi.ajp.161.8.1390 15285964

[mpr1579-bib-0032] Olff, M. (2015). Mobile mental health: A challenging research agenda. European Journal of Psychotraumatology, 6, 27882 10.3402/ejpt.v6.27882 25994025 PMC4439418

[mpr1579-bib-0033] Owen, J. E. , Jaworski, B. K. , Kuhn, E. , Makin‐Byrd, K. N. , Ramsey, K. M. , & Hoffman, J. E. (2015). mHealth in the wild: Using novel data to examine the reach, use, and impact of PTSD coach. JMIR Ment Health, 2(1). e7. doi:10.2196/mental.3935 PMC4607374 26543913

[mpr1579-bib-0034] Possemato, K. , Kuhn, E. , Johnson, E. M. , Hoffman, J. E. , & Brooks, E. (2016). Development and refinement of a clinician intervention to facilitate primary care patient use of the PTSD Coach app. Translational Behavioral Medicine. 10.1007/s13142-016-0393-9 PMC5352634 27234150

[mpr1579-bib-0035] Price, M. , Kuhn, E. , Hoffman, J. E. , Ruzek, J. , & Acierno, R. (2015). Comparison of the PTSD Checklist (PCL) administered via a mobile device relative to a paper form. Journal of Traumatic Stress, 28(5), 480–483. 10.1002/jts.22037 26375277

[mpr1579-bib-0036] Price, M. , Ruggiero, K. J. , Ferguson, P. L. , Patel, S. K. , Treiber, F. , Couillard, D. , & Fahkry, S. M. (2014). A feasibility pilot study on the use of text messages to track PTSD symptoms after a traumatic injury. General Hospital Psychiatry, 36(3), 249–254. 10.1016/j.genhosppsych.2014.02.004 24636721 PMC4090249

[mpr1579-bib-0037] Price, M. , Sawyer, T. , Harris, M. , & Skalka, C. (2016). Usability evaluation of a mobile monitoring system to assess symptoms after a traumatic injury: A mixed‐methods study. JMIR Ment Health, 3(1). e3. doi:10.2196/mental.5023 PMC4726868 26753673

[mpr1579-bib-0038] Price, M. , Yuen, E. K. , Goetter, E. M. , Herbert, J. D. , Forman, E. M. , Acierno, R. , & Ruggiero, K. J. (2014). mHealth: A mechanism to deliver more accessible, more effective mental health care. Clinical Psychology & Psychotherapy, 21(5), 427–436. 10.1002/cpp.1855 23918764 PMC3926903

[mpr1579-bib-0039] Roberts, N. P. , Kitchiner, N. J. , Kenardy, J. , & Bisson, J. (2009). Multiple session early psychological interventions for the prevention of post‐traumatic stress disorder. Cochrane Database of Systematic Reviews, 3. CD006869. doi:10.1002/14651858.CD006869.pub2 19588408

[mpr1579-bib-0040] Shalev, A. Y. , Ankri, Y. L. , Peleg, T. , Israeli‐Shalev, Y. , & Freedman, S. (2011). Barriers to receiving early care for PTSD: Results from the Jerusalem trauma outreach and prevention study. Psychiatric Services, 62(7), 765–773. 10.1176/ps.62.7.pss6207_0765 21724790

[mpr1579-bib-0041] Shalev, A. Y. , Freedman, S. , Peri, T. , Brandes, D. , Sahar, T. , Orr, S. P. , & Pitman, R. K. (1998). Prospective study of posttraumatic stress disorder and depression following trauma. The American Journal of Psychiatry, 155(5), 630–637. 10.1176/ajp.155.5.630 9585714

[mpr1579-bib-0042] Spitzer, B. , Gibbon, R. L. , Janet, M. , & Janet, W. (1996). Structured Clinical Interview for DSM‐IV Axis I Disorders—Patient edition (SCID I/P, version 2.0). New York, NY: American Psychiatric Press.

[mpr1579-bib-0043] van der Meer, C. A. , Bakker, A. , Smit, A. S. , van Buschbach, S. , den Dekker, M. , Westerveld, G. J. , … Olff, M. (2016). Gender and age differences in trauma and PTSD among Dutch treatment‐seeking police officers. The Journal of Nervous and Mental Disease. 10.1097/nmd.0000000000000562 27434192

[mpr1579-bib-0044] Van Groenestijn, M. A. C. , Akkerhuis, G. W. , Kupka, R. W. , Schneider, N. , & Nolen, W. A. (1999). Structured clinical interview for Axis I disorders (SCID‐I; Dutch version). Lisse, The Netherlands: Swets & Zeitlinger.

[mpr1579-bib-0045] Weathers, F. W. , Blake, D. D. , Schnurr, P. P. , Kaloupek, D. G. , Marx, B. P. , & Keane, T. M. (2013). The clinician‐administered PTSD scale for DSM‐5 (CAPS‐5) [Interview]. Retrieved from the National Center for PTSD website: http://www.ptsd.va.gov/

[mpr1579-bib-0046] Weathers, F. W. , Litz, B. T. , Keane, T. M. , Palmieri, P. A. , Marx, B. P. , & Schnurr, P. P. (2013). The PTSD checklist for DSM‐5 (PCL‐5) [Scale]. Retrieved from the National Center for PTSD website: http://www.ptsd.va.gov/.

[mpr1579-bib-0047] Wortmann, J. H. , Jordan, A. H. , Weathers, F. W. , Resick, P. A. , Dondanville, K. A. , Hall‐Clark, B. , … Litz, B. T. (2016). Psychometric analysis of the PTSD Checklist‐5 (PCL‐5) among treatment‐seeking military service members. Psychological Assessment, 28(11), 1392–1403. 10.1037/pas0000260 26751087

